# Increased Expression of Gp96 by HBx-Induced NF-κB Activation Feedback Enhances Hepatitis B Virus Production

**DOI:** 10.1371/journal.pone.0065588

**Published:** 2013-06-11

**Authors:** Hongxia Fan, Xiaoli Yan, Yu Zhang, Xiaojun Zhang, Yanzhou Gao, Yaxing Xu, Fusheng Wang, Songdong Meng

**Affiliations:** 1 CAS Key Laboratory of Pathogenic Microbiology and Immunology, Institute of Microbiology, Chinese Academy of Sciences (CAS), Beijing, China; 2 Beijing Institute of Infectious Diseases, Beijing 302 Hospital, Beijing, China; UC Irvine Medical Center, United States of America

## Abstract

Elevated expression of heat shock protein gp96 in hepatitis B virus (HBV)-infected patients is positively correlated with the progress of HBV-induced diseases, but little is known regarding the molecular mechanism of virus-induced gp96 expression and its impact on HBV infection. In this study, up-regulation of gp96 by HBV replication was confirmed both *in vitro* and *in vivo*. Among HBV components, HBV x protein (HBx) was found to increase gp96 promoter activity and enhance gp96 expression by using a luciferase reporter system, and western blot analysis. Further, we found that HBx-mediated regulation of gp96 expression requires a NF-κB cis-regulatory element on the gp96 promoter, and chromatin immunoprecipitation results demonstrated that HBx promotes the binding of NF-κB to the gp96 promoter. Significantly, both gain- and loss-of-function studies showed that gp96 enhances HBV production in HBV-transfected cells and a mouse model based on hydrodynamic transfection. Moreover, up-regulated gp96 expression was observed in HBV-infected patients, and gp96 levels were correlated with serum viral loads. Thus, our work demonstrates a positive feedback regulatory pathway involving gp96 and HBV, which may contribute to persistent HBV infection. Our data also indicate that modulation of gp96 function may represent a novel strategy for the intervention of HBV infection.

## Introduction

Hepatitis B virus (HBV) infection is a major global public health problem. Over 350 million individuals are chronically infected, and patients with chronic hepatitis B (CHB) are at high risk of developing cirrhosis, liver failure, and hepatocellular carcinoma (HCC) [1]. HBV, the prototypic member of the *Hepadnaviridae* family, is a small enveloped hepatotropic DNA virus of ∼3.2 kb in length that relies heavily on host cellular machinery to complete its replication and pathogenesis. The HBV genome contains four overlapping open reading frames that efficiently encode several overlapping viral proteins, including the pol, core, HBe, Pre-S1, S2, S and X proteins. A large number of host factors are critical for multiple steps in the HBV replication cycle [2]–[8], among which heat shock proteins have gained much attention due to their enigmatic chaperone function in DNA replication, viral assembly, and other processes. For instance, hsp90 (and its co-chaperones p23 and p50/CDC37), hsp70, and hsp40 may form an HSP complex that interacts with the virus pol and thereby facilitates formation of the ribonucleoprotein (RNP) complex between pol and the pregenomic RNA (pgRNA) [2], [8]. Hsp60 is required for maturation of HBV pol to an active state [5]. In addition, hsp70, hsp40, and GRP78/BiP are involved in HBV envelop protein translocation and HBV morphogenesis [3], [6]. Nevertheless, a detailed understanding of the possible reciprocal effects of HSPs and HBV, as well as their clinical relevance in HBV infection, is lacking.

Heat shock protein gp96, a member of hsp90 family, predominantly resides within the lumen of the endoplasmic reticulum (ER) and is constitutively expressed in all cell types [9]. As one of the most abundant proteins in the ER, gp96 is able to associate with client proteins and guide their maturation and assembly. However, the chaperone activity of gp96 is rather selective, which is in contrast with its cytosolic counterpart hsp90, as well as other HSPs (e.g., GRP78) that may remodel and activate hundreds of client proteins [10], [11]. To date, only a handful of confirmed clients for gp96 have been identified, which include cell surface ligands and receptors (e.g., certain Toll-like receptors [12], [13], integrins [12], platelet glycoprotein Ib-IX-V [14], insulin-like growth factors [15], [16]), and proteins and enzymes localized in the secretory pathway (e.g., a disintegrin-like and metalloprotease domain with thrombospondin type 1 motifs 9 (ADAMTS9) [17] and immunoglobulin [18]).

Previously, our lab found that the gp96 expression in liver tissues of patients with CHB is significantly increased compared to that of uninfected controls, and the extent of elevated gp96 expression is significantly associated with the disease progression of HBV infection [19], [20]. Similar results were also observed in other studies [21], [22]. However, the mechanisms involved in gp96 up-regulation by viral infection and whether and how gp96 up-regulation may be involved in the regulation of viral replication are unknown.

Given its cellular abundance and unique restriction to bind client proteins, in the present study we aimed to explore the mechanism of gp96 up-regulation by HBV infection and the potential role of gp96 in regulating viral replication. We demonstrated that HBx-mediated NF-κB activation stimulates gp96 expression, with the elevated gp96 in turn enhancing HBV production. These results suggest that a positive feedback loop involving gp96 and HBV may provide a favorable environment for persistent HBV infection.

## Materials and Methods

### Ethics Statement

For human subjects: written informed consent was provided by all study participants. The study protocol was approved by the ethics committee of Beijing 302 Hospital. The study of mice was in strict accordance with “the regulation of the Institute of Microbiology, Chinese Academy of Sciences of Research Ethics Committee,” The protocol was approved by the committee (Permit Number: PZIMCAS2012004).

### Patients and Tissue Specimens

Paraffin-embedded liver sections from fifty nine patients with CHB were collected from Beijing 302 Hospital between January 2009 and January 2010. The diagnosis standards for CHB were complied with the diagnostic criteria of the 2005 Guideline of Prevention and Treatment for Chronic Hepatitis B issued by Chinese Society of Infectious Diseases and Chinese Society of Hepatology, Chinese Medical Association. All patients had chronic HBV infection with serum HBV surface antigen (HBsAg) positivity for >6 months and exhibited symptoms of hepatitis and abnormal hepatic function. The CHB patients were classified into HBeAg positive and negative group according to their serum HBeAg status. No patients received anti-HBV agent, corticosteroid or immunosuppressive agents 6 months before blood and liver sampling. Patients with concurrent infection with hepatitis C virus, hepatitis D virus, human immunodeficiency virus and other causes of liver disease were excluded. 10 matched healthy human liver specimens were obtained from health donors livers used for transplantation. The clinical characteristics of the studied subjects are listed in Table 1.

**Table 1:** Clinical characteristics of the population enrolled in this study.

Parameter	Healthy control	CHB
Case	10	59
Gender (male/female)	6/4	35/24
Age, year (range)	24 (18–40)	28 (18–45)
HBsAg positive	0	59
HBeAg positive	0	20
ALT, U/L (range)	23 (8–36)	276 (35–926)
AST, U/L (range)	26 (10–39)	150 (23–473)
HBV DNA, copies/ml (range)	ND	108,237,288 (15,000,000–276,000,000)

ND, not determined.

### Reagents and Antibodies

The gp96-specific small interfering RNA (siRNA), ERK1- and ERK2- specific siRNA, and control siRNA (specific for luciferase) were designed and synthesized by RiboBio Co., Ltd. (Guangzhou, China). The siRNA sequences for gp96 and luciferase were 5′-GCAAGGACATCTCTACAAA-3′ and 5′-CTTACGCTGAGTACTTCGA-3′, respectively.

The following reagents and antibodies were obtained as indicated: ammonium pyrrolidinedithiocarbamate (PDTC), a NF-κB inhibitor was purchased from Beyotime Company (Jiangsu, China); Anti-gp96, anti-p65 and anti-p50 (Santa Cruz Biotechnology, CA), anti-hsp90 (Bioworld Technology, Minneapolis, USA), anti-hsp70 (gifted by Prof. Xin Ye, IMCAS, Beijing, China), anti-HBcAg (DAKO, Carpinteria, CA), anti-HBs antibody (Virostat, Portland, ME), the antibodies against Myc tag, mouse anti-human actin antibody, mouse anti-human GAPDH antibody and horseradish peroxidase-conjugated secondary antibodies (Zhongshan Goldenbridge Biotechnology, Beijing, China); the ECL-Plus chemiluminescence system (Applygen Technologies, Beijing, China).

### Cell Culture and Transfection

The human hepatoma cell line Huh7 and Chang cell line were obtained from the ATCC (Manassas, VA, USA). Huh7 cells were cultured in Dulbecco’s modified Eagle’s medium (DMEM) supplemented with 10% fetal bovine serum (Gibco, NY, USA). The human Chang cell line was grown in RPMI 1640 with 10% fetal bovine serum. All cells were incubated at 37°C in an atmosphere containing 5% CO_2_. All transfections were carried out with Lipofectamine 2000 (Invitrogen) or GenJet**™** (SignaGen) as recommended by the manufacturers.

### Plasmids and Recombinant Adenovirus

pHBV containing 1.3 copies of full-length HBV genomic sequence was maintained in the lab. The HBV-luciferase plasmid pHBV-Luc was kindly provided by Yosef Shaul (The Weizmann Institute of Science, Israel). pcDNA3.1-preS1, pcDNA3.1-preS2 and pcDNA3.1-HBs plasmids were generously provided by Dr. Yi-ping Zhu (IMCAS, Beijing, China). The other individual viral structural genes of HBV (pcDNA3.1-HBc, pcDNA3.1-HBe, pcDNA3.1-HBx, pcDNA3.1-HBp) were constructed in the lab. pcDNA3.1-preS1 vector carries preS1 gene with mutations in the translational start sites of the overlapping ORFs that ablate the preS2 and HBs expression. pcDNA3.1-preS2 vector carries preS2 gene with a mutation that ablates the HBs expression. pcDNA3.1-HBp vector carries pol gene with mutations that ablate the expression of large, middle, and small surface proteins. The expression of the specific proteins was confirmed by western blotting analysis. The plasmids pcDNA3.1-p50 and pcDNA3.1-p65 were obtained from Prof. Xin Ye (IMCAS, Beijing, China). A 891-bp gp96 promoter construct, corresponding to the sequence from −799 to +79 of the 5′-flanking region of the gp96 gene, was generated by double restriction enzyme digestion of pGL3-PromTRA1 (gifted by Prof. Carine Michiels, University of Namur, Namur, Belgium) and subcloning into the pGL3-basic vector. Point mutation in the gp96 promoter construct was performed with NEWPEP Site-Directed Mutagenesis Kit (Beijing NewPep Biotech Co., Ltd.) with mutated primers designed as described [23]. The full length gp96 gene was cloned into bacterial expression vector pGEX-6p-1 and mammalian expression vector pcDNA3.1-Flag, respectively.

Adenovirus ad-CMV and ad-gp96 were created using the AdMax™ system (Microbix, Toronto, Canada), and the virus were propagated and purified with ViraTrap™ Adenovirus Purification Miniprep Kit (Biomiga, USA). Virus titer was determined by plaque assay in HEK293 cells.

### Luciferase Reporter Assays

Cells were co-transfected with the promoter luciferase reporter plasmid and the pRL-TK plasmid. 48 h after transfection, cells were harvested and detected with the Dual Luciferase Reporter Assay System (Promega, Madison, WI).

### Viral RNA, DNA Extraction and Analysis

HBV replicative intermediates and HBV transcripts were extracted and analyzed with Southern blot and northern blot, respectively, or real-time PCR as described previously [24]. Core particle-associated HBV DNA from cell culture supernatant or blood was extracted and quantified with real-time PCR as described [25]. The primers used are as follows: pgRNA(forward), 5′-TCTTGCCTTACTTTTGGAAG-3′ (nt 2216–2235) and pgRNA(reverse), 5′-AGTTCTTCTTCTAGGGGACC-3′ (nt 2361–2380); total RNA(forward) designed in HBx region, 5′-ACGTCCTTTGTTTACGTCCCGT-3′ (nt 1414–1435) and total RNA (reverse), 5′-CCCAACTCCTCCCAGTCCTTAA-3′ (nt 1744–1723).

### Real-time PCR

Total RNA was extracted with Trizol Reagent, and quantified by real-time PCR using the SYBR Green Premix Reagent (Takara Bio Inc., Shiga, Japan) with a GAPDH internal control for normalization.

### Detection of HBsAg and HBeAg

The expression levels of HBsAg and HBeAg were measured by ELISA as described previously [24].

### Cesium Chloride Density Gradient Centrifugation of Extracellular HBV Particles

Culture supernatants from Chang cells were collected at day 3 post-transfection and concentrated with Amicon Ultra-15 centrifugal filter unit with an Ultracel-50 membrane (Millipore Corp., Bedford, MA, USA). The concentrated supernatants were fractionated by isopycnic cesium chloride gradient ultracentrifugation [26]. Fractions of 400 µl were collected and weighed to obtain the density values. After dialysis against TNE buffer (10 mM Tris [pH8.0], 1 mM EDTA, 150 mM NaCl), they were analyzed by immunoassay for HBsAg. Fractions corresponding to the virions were pooled for DNA extraction and further analyzed with real-time PCR.

### Chromatin Immunoprecipitation Assay (CHIP)

Huh7 cells transfected with pcDNA3.1-HBx or mock plasmids were cross-linked with 1% formaldehyde and sonicated to small fragments. Then the supernatant was incubated with an antibody against p65, p50 or an isotype control IgG overnight at 4°C. The immunoprecipitated DNA was extracted and subjected to PCR using gp96 promoter-specific primers: 5′-ACGTTGCATGCCGG GAGCTGTAGT-3′ (forward) and 5′-GCACGACTTTCGCGGCCAGCTTTC-3′ (reverse).

### Electrophoretic Mobility Shift Assay (EMSA)

Nuclear extracts were prepared from Huh7 cells transfected with relative expression vectors. Biotin end-labeled double-stranded DNA probes were generated by annealing complementary oligonucleotides. The oligonucleotide sequences were 5′-GTTCTAGGGGATTTGCAACCTCTC-3′ and 5′-GAGAGGTTGCAAATCCCCTAGAAC-3′. EMSA was performed using LightShift Chemiluminescent EMSA Kit (Pierce) according to the manufacturer’s protocol. Briefly, 4 µg nuclear extracts were incubated for 20 min at room temperature in a volume of 20 µl containing 40 fmol biotin labeled probes, 50 ng poly(I:C) and 2 µl 10×binding buffer. For competition experiment, 100-fold of unlabeled wild type probes were added prior to the labeled probe addition. For p65 depletion experiment, 1 µg polyclonal antibody against p65 were added to nuclear extract and incubated for 20 min before adding to the binding reaction. Samples were then separated on a 6% non-denaturing polyacrylamide gel and visualized by chemiluminescence.

### Animal Studies

Female BALB/c mice were purchased from Peking University, Beijing. Female HBV transgenic BALB/c mice were purchased from Transgenic Engineering Lab, Infectious Disease Center (Guangzhou, China). In hydrodynamic mouse model, a total of 5 µg pHBV and 1×10^9 ^pfu adenovirus ad-CMV or ad-gp96 were injected into the mouse tail vein via hydrodynamic injection. Three days after injection, mice were sacrificed. The sera were measured for HBsAg, HBeAg and HBV DNA levels. Liver samples were examined for intracellular HBV DNA replicative intermediates with real-time PCR. HBV core protein (HBcAg) was detected with immunohistochemical staining assay.

### Immunohistochemistry (IHC)

Immunohistochemical staining was performed as previously described [24]. Gp96 staining was categorized as gp96 high (score 3+), medium (score 2+) or low (score 0/1+) expression according to the staining intensity and immunoreactive cell percentage according to the widely used scoring method (slightly modified) [27]. The scoring was described as follows: Score 0/1+, no staining or faint and incomplete cytoplasmic staining of hepatocytes; Score 2+, weak to moderate complete cytoplasmic staining in at least 50% of hepatocytes; Score 3+, strong complete cytoplasmic staining in >50% of hepatocytes. The assessments were scored by two independent observers in a blinded manner.

### Statistical Analysis

All data were analyzed using SPSS software (SPSS Science, Chicago, IL). Student’s t-test or Pearson’s χ test was applied to compare groups. The degree of association between variables was determined by Spearman’s non-parametric correlation rank. Results were presented as means ± standard deviations. A P value less than 0.05 is considered significant.

## Results

### HBx Protein Induces gp96 Expression

**Figure 1:**
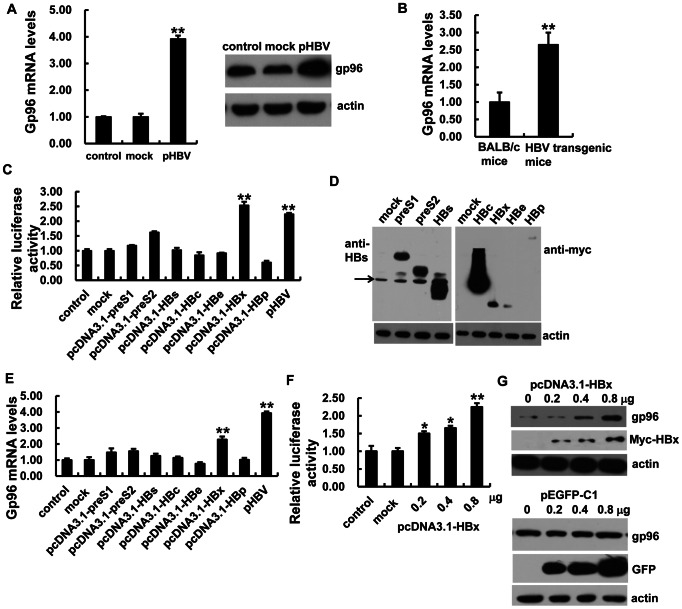
HBx protein induces gp96 expression. (A) Real-time PCR and western blot analysis of gp96 mRNA and protein levels in Huh7 cells transfected with the HBV expression vector pHBV or a GFP expression vector pEGFP-C1 (mock) or the empty vector pcDNA3.1 as a control. (B) The gp96 mRNA levels in liver were detected in HBV transgenic BALB/c mice and BALB/c mice as controls. (C) Huh7 cells were co-transfected with the gp96 promoter luciferase reporter plasmid and pHBV, the indicated individual HBV protein-expressing plasmids, or pcDNA3.1 as a control or pEGFP-C1 as a mock. 48 h after transfection, firefly luciferase and Renilla luciferase activities were measured, and the luciferase activity was normalized to Renilla luciferase. The luciferase activity of mock-transfected cells was designated as 1.0. (D, E) Huh7 cells were transfected with the indicated individual HBV protein-expressing plasmids, pHBV, or pcDNA3.1 as a control or pEGFP-C1 as a mock. The expression of HBV preS1, preS2 and HBs proteins were determined by western blotting using anti-HBs antibody, and the expression of myc-tagged HBc, HBx, HBe and pol (HBp) proteins was determined by western blotting using anti-Myc antibody. The arrow indicates a non-specific band (D). The gp96 mRNA levels were analyzed by real-time PCR after 48 h (E). (F) Huh7 cells were co-transfected with the gp96 promoter luciferase reporter plasmid and the indicated dose of HBx expression plasmid pcDNA3.1-HBx, pcDNA3.1 as a control or pEGFP-C1 as a mock. The relative luciferase activity was then determined. (G) Huh7 cells were transfected with the indicated amount of pcDNA3.1-HBx or pEGFP-C1 as a mock, and gp96 protein levels were determined 48 h post-transfection. The expression of myc-tagged HBx was detected by western blotting using anti-Myc. Data are presented as the means ± SD from three independent experiments. *P<0.05 and **P<0.01 compared to mock.

To understand how HBV infection might influence gp96 protein levels, we first tested if viral factors directly affect gp96 expression. Transfection with the HBV expression vector pHBV, which contains 1.3 copies of full-length HBV genome, resulted in an approximately 3-fold increase of both gp96 mRNA and protein levels in Huh7 cells (Fig. 1A). Moreover, HBV transgenic BALB/c mice, which are generated with a terminally redundant viral DNA construct (HBV 1.3) and were positive for serum HBsAg, as well as virus DNA and HBcAg in liver, were then used to determine if HBV replication directly affects gp96 expression *in vivo*. We found that the gp96 mRNA in HBV transgenic mice were higher than those in the parental BALB/c mice (Fig. 1B).

Next, we explored HBV viral factors that may account for gp96 up-regulation. Huh7 cells were co-transfected with a gp96 promoter luciferase reporter plasmid and pHBV or individual HBV protein-expressing plasmids encoding Pre-S1, S2, HBc, HBe, HBx, or pol (HBp). As shown in Fig. 1C, aside from the HBV expression vector pHBV, the gp96 promoter activity was markedly stimulated by transfection with pcDNA3.1-HBx. The expression of individual HBV proteins was confirmed by western blotting as shown in Fig. 1D. Real-time PCR analysis of gp96 mRNA levels also revealed a similar result (Fig. 1E). Moreover, up-regulation of gp96 promoter activity (Fig. 1F), and protein levels (Fig. 1G) followed a dose-response curve after treatment with increasing amounts of HBx. In parallel, over-expression of an irrelevant protein (GFP) had no obvious effect on gp96 expression.

### HBx-induced gp96 Expression is Mediated Through NF-κB Activation

**Figure 2:**
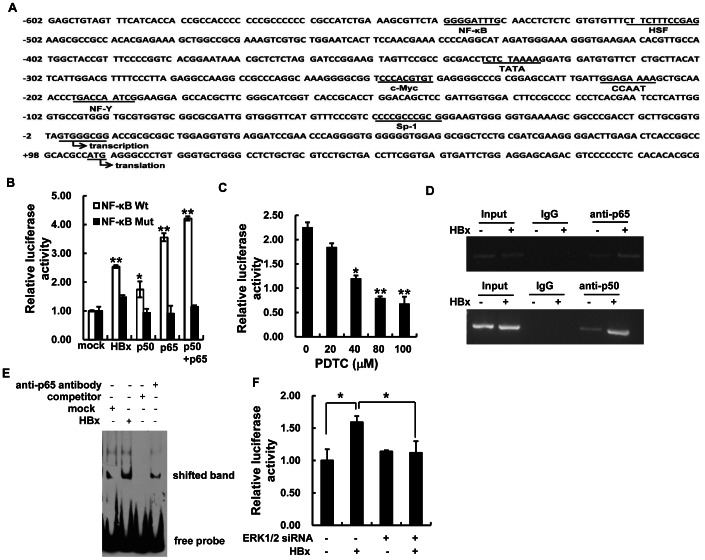
HBx up-regulates the expression of gp96 by activating NF-κB. (A) Sequence analysis of the human gp96 promoter using the online TFSEARCH program. The putative binding sites for several cis-acting elements are indicated. (B) Huh7 cells were co-transfected with the gp96 promoter luciferase reporter plasmid with a wild type or mutated NF-κB binding site and pcDNA3.1-HBx, pcDNA3.1-p50 and pcDNA3.1-p65 as the positive controls, or pEGFP-C1 as a mock. The relative luciferase activity was determined 48 h after transfection. The luciferase activity of the mock-transfected group was set as 1.0. (C) Huh7 cells were treated with the indicated concentration of PDTC after co-transfection with pcDNA3.1-HBx and the gp96 promoter luciferase reporter plasmid. The luciferase activity in untreated cells was used as a control. (D) CHIP analysis was performed with cross-linked cell fragments of Huh7 cells transfected with pcDNA3.1-HBx or mock plasmids. The immunoprecipitated DNA was analyzed by PCR using gp96 promoter-specific primers. (E) EMSA analysis was performed with nuclear extracts of Huh7 cells transfected with pcDNA3.1-HBx or mock plasmids. (F) Huh7 cells were co-transfected with pcDNA3.1-HBx, ERK siRNA and the gp96 promoter luciferase reporter plasmid. The relative luciferase activity was determined 48 h after transfection. The luciferase activity of the mock-transfected group was set as 1.0. Data are presented as the means ± SD from three independent experiments. *P<0.05 and **P<0.01 compared to the mock or control.

To investigate the underlying basis for HBx-mediated gp96 up-regulation, the gp96 promoter was subjected to bioinformatics analysis (http://www.cbrc.jp/research/db/TFSEARCH.html), revealing that there is a putative NF-κB binding site in the promoter region (nt −533 to −542) (Fig. 2A). As HBx is able to transactivate the NF-κB transcription factor, we investigated whether the HBx-induced NF-κB activation is involved in the regulation of gp96 expression. As shown in Fig. 2B, the gp96 promoter activity of a wild type but not mutant in NF-κB binding site was significantly increased in pcDNA3.1-HBx transfected cells compared to the mock. Similar results were observed for the p50 and p65 subunits of NF-κB, as expected. Moreover, activation of the gp96 promoter by HBx could be blocked by the NF-κB inhibitor PDTC (Fig. 2C). CHIP assay also showed that HBx induced a large increase in the association of NF-κB subunits p65 and p50 with the gp96 promoter (Fig. 2D). In addition, EMSA experiment was carried out with HBx transfected cell nuclear extracts and the biotin-labeled oligonucleotide probe containing the p65-binding sequence from the gp96 promoter. The results showed that HBx increased p65 binding to the gp96 promoter (Fig. 2E), indicating that HBx up-regulates gp96 transcription by activation of the NF-κB. The specificity of the complexes was further confirmed by the p65 antibody or unlabeled probe incubation experiment.

HBx has been shown to activate NF-κB through ERK pathway [28]–[30]. To further demonstrate that HBx increases gp96 expression by the ERK/NF-κB pathway, Huh7 cells were treated with pcDNA3.1-HBx and ERK siRNA, as indicated in Fig. 2F. The activation effect of HBx on gp96 promoter activity was largely abolished by ERK RNAi. Taken together, these results suggest that the induced up-regulation of gp96 expression occurs via activation of ERK/NF-κB pathway by HBx, leading to the subsequent binding of NF-κB subunits p65 and p50 to the gp96 promoter.

### Gp96 Enhances HBV Production

**Figure 3:**
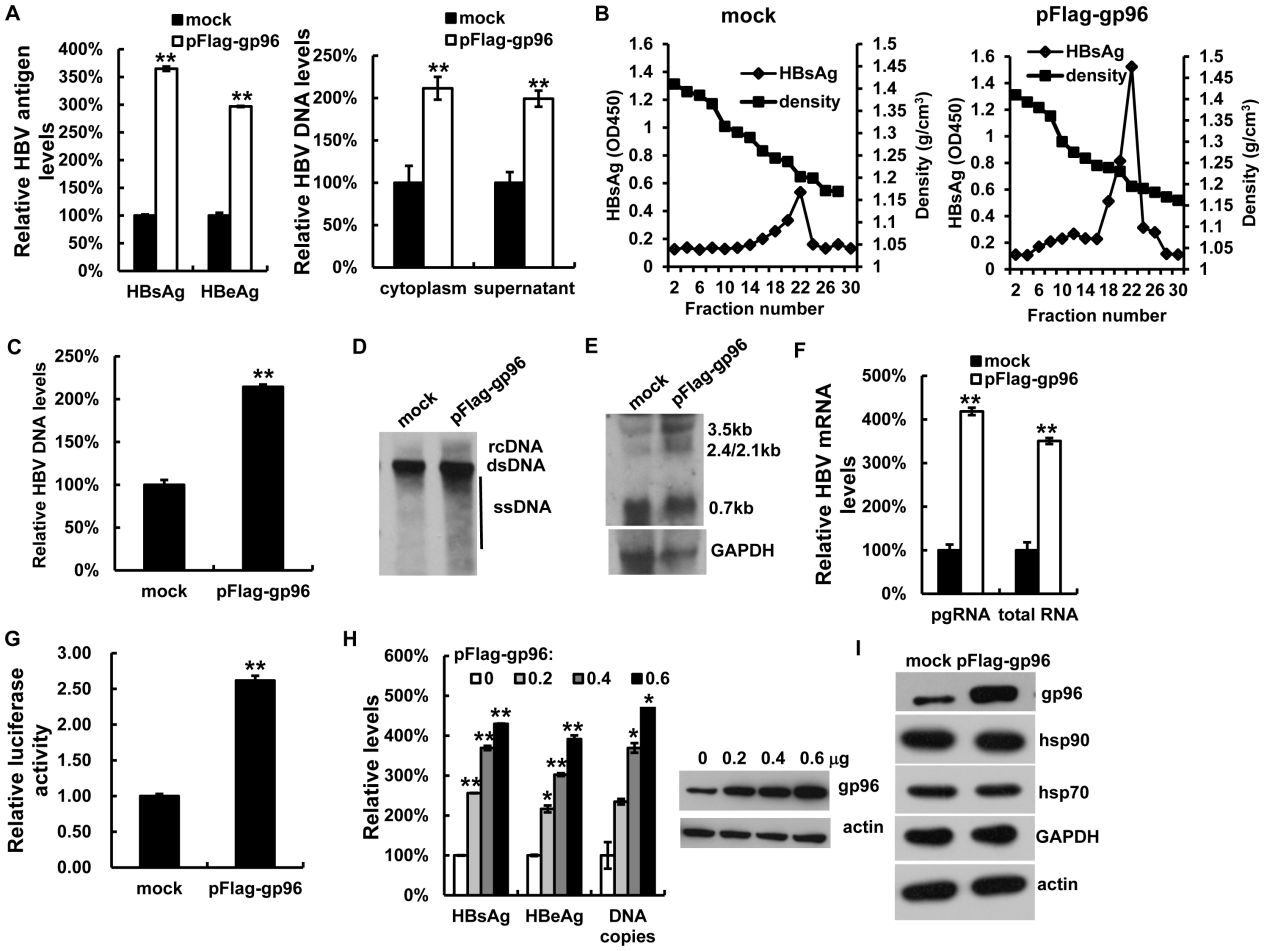
Over-expression of gp96 increases HBV production *in vitro*. Chang cells were co-transfected with pFlag-gp96 or pEGFP-C1 (mock) and pHBV. (A) The levels of HBsAg and HBeAg in the culture supernatant were measured by ELISA (left panel), and the HBV DNA levels in the culture supernatant and cytoplasm were quantified by real-time PCR (right panel). (B, C) The culture supernatants were subjected to isopycnic centrifugation in a gradient of 20 to 50% (wt/vol) cesium chloride. Fractions of 400 µl were collected, dialyzed and assayed for the HBsAg by ELISA (B). The fractions corresponding to the virion particles (density: 1.22–1.24 g/cm^3^) were pooled for DNA extraction, and analyzed by real-time PCR (C). (D) The cytoplasmic HBV DNA was detected by Southern blotting. rcDNA, relaxed circular; dsDNA, double-stranded; ssDNA, single-stranded HBV DNA. (E and F) HBV pgRNA and total RNA levels were determined by northern blotting (E) and real-time PCR (F). (G) Chang cells were co-transfected with pHBV-luc and pFlag-gp96. At 48 h post-transfection, the relative luciferase activity was determined. (H) Chang cells were co-transfected with pHBV along with increasing amounts of pFlag-gp96. 48 h post-transfection, the levels of HBsAg, HBeAg, and HBV DNA copies in the supernatant were analyzed. (I) The protein levels of gp96, hsp90, hsp70, GAPDH and actin were determined by western blotting. Data are presented as the means ± SD from three independent experiments. *P<0.05 and **P<0.01 compared to mock.

Next, we examined the effect of gp96 on HBV production. As transfection of the gp96 expression plasmids could not lead to an obvious increase in gp96 protein levels in Huh7 cells, we chose Chang cells for gp96 over-expression study. As shown in Fig. 3A, compared to mock (GFP), over-expression of gp96 by transfection of the gp96 expression plasmid pFlag-gp96 in Chang cells led to an approximately 2- to 3- fold increase in HBsAg and HBeAg expression in the culture supernatant (both P<0.01), as well as an increase in HBV DNA levels in the cytoplasm and cell culture supernatant. The gradient centrifugation analysis of cell supernatant showed much higher levels (more than 2-fold) of HBV particles (Fig. 3B) and viral DNA (Fig. 3C) in the gradient fractions corresponding to the known density of HBV Dane particles (around 1.24g/cm^3^) in gp96-transfected cells relative to mock. A significant increase in cytoplasmic HBV DNA replicative intermediates was observed in gp96-transfected cells by Southern blot analysis (Fig. 3D). Further, compared to the mock, the HBV pgRNA and total mRNA levels were consistently 2- to 3-fold greater in gp96-transfected cells, as shown by northern blot analysis and real-time PCR (Fig. 3E and 3F). To further investigate the effect of gp96 on HBV transcription, we performed HBV promoter luciferase assay. Chang cells were co-transfected with pFlag-gp96 and an HBV-luciferase plasmid (pHBV-Luc), an HBV expressing construct containing a luciferase ORF under the HBV core promoter. Transfection of pFlag-gp96 led to an increase of HBV core promoter activity by around 2-fold (Fig. 3G).Transfection of Chang cells with increasing amounts of pFlag-gp96 increased HBV production in a dose-dependent manner (Fig. 3H). Meanwhile, gp96 over-expression did not significantly affect levels of the control proteins (Fig. 3I), indicating that the effect of gp96 on HBV production was specific.

**Figure 4:**
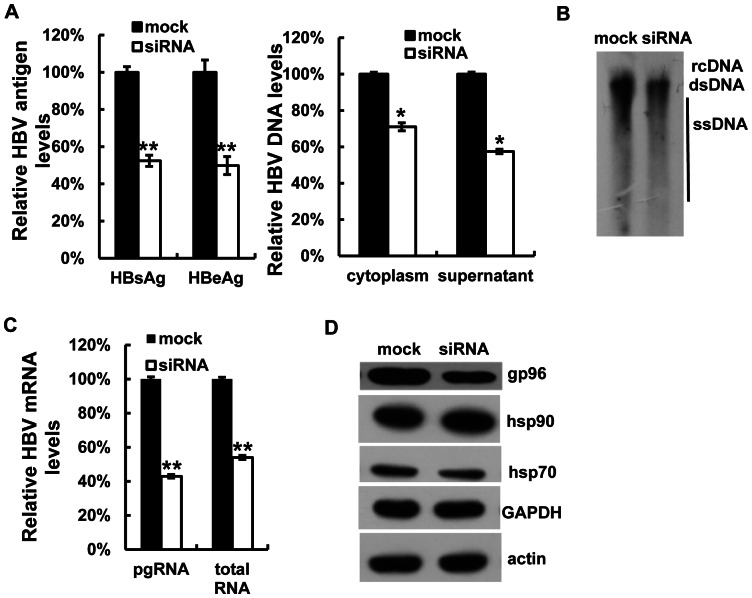
Suppression of gp96 expression decreases HBV production *in vitro*. Huh7 cells were co-transfected with pHBV and gp96-specific siRNA or luciferase-specific siRNA as mock. The secretion of HBsAg and HBeAg was determined by ELISA (A). The HBV DNA levels in the supernatant and cytoplasm were determined by real-time PCR (A) and Southern blotting (B). The HBV pgRNA and total RNA were analyzed by real-time PCR (C). The protein levels of gp96, hsp90, hsp70, GAPDH and actin were determined by western blotting (D). Data are presented as the means ±SD from three independent experiments. *P<0.05 and **P<0.01 compared to the mock.

Conversely, when Huh7 cells were transfected with gp96 siRNA, marked decreases in HBsAg and HBeAg expression in culture supernatant, as well as HBV DNA levels in the cytoplasm and cell culture supernatant, were observed compared to the mock (Fig. 4A). Silencing of gp96 caused pronounced reductions in HBV DNA replicative intermediates, viral pgRNA, and total mRNA levels (Fig. 4B and 4C). The silencing effect of gp96 siRNA and its specificity on HBV proteins were confirmed by western blot analysis of gp96, hsp70, hsp90 and GAPDH levels (Fig. 4D).

In the meantime, CCK-8 assays were performed to determine the potential effect of gp96 over-expression or knock-down on cell proliferation. Transfection of Chang cells with pFlag-gp96 induced an ∼30% increase of cell proliferation 72 h post-transfection, while for Huh7 cells, only a 10% decrease in cell proliferation was observed 72 h after transfection of gp96 siRNA, compared to the mock (data not shown). These results suggest that modulation of HBV production by gp96 is not due to its impact on cell proliferation.

**Figure 5:**
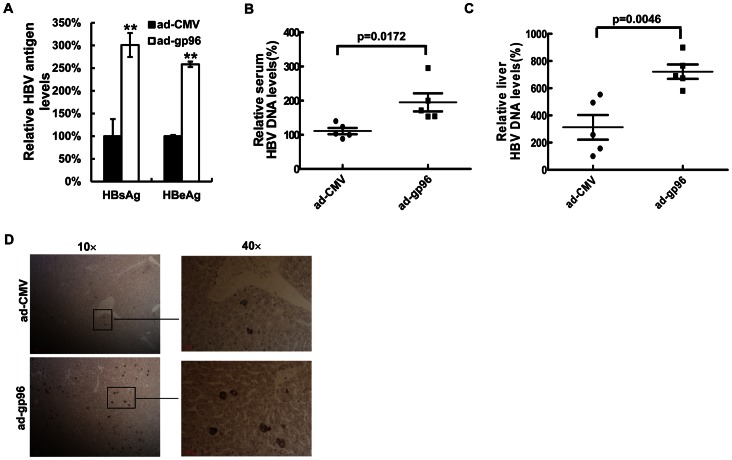
Gp96 promotes HBV production in mice. BALB/c mice were hydrodynamically co-injected with 5 µg pHBV and 1×10^9^ pfu adenovirus ad-gp96 (n = 5) or ad-CMV as a mock (n = 5). Three days later, the HBsAg and HBeAg levels in serum were measured by ELISA (A). HBV DNA levels in the serum (B) and liver (C) were determined by real-time PCR. HBcAg expression in the liver was examined by immunohistochemistry (D). *P<0.05 and **P<0.01 compared to the mock. Three independent experiments were performed with similar results.

We further investigated the effect of gp96 on HBV production *in vivo* in BALB/c mice that were hydrodynamically injected with pHBV. In concert with the *in vitro* results, adenoviral over-expression of gp96 by injection of ad-gp96 led to an approximately 2-fold increase in HBsAg and HBeAg serum levels (Fig. 5A). Significant increases in HBV DNA levels in serum (Fig. 5B) and liver (Fig. 5C) were also observed compared to the mock. Immunohistochemical staining results revealed that the number of HBcAg-positive cells in ad-gp96-treated mice is much greater than in mock-treated mice (Fig. 5D).

### Gp96 Expression Levels in the Liver of HBV-infected Patients are Associated with Viral Loads and Serum ALT/AST Levels

**Figure 6:**
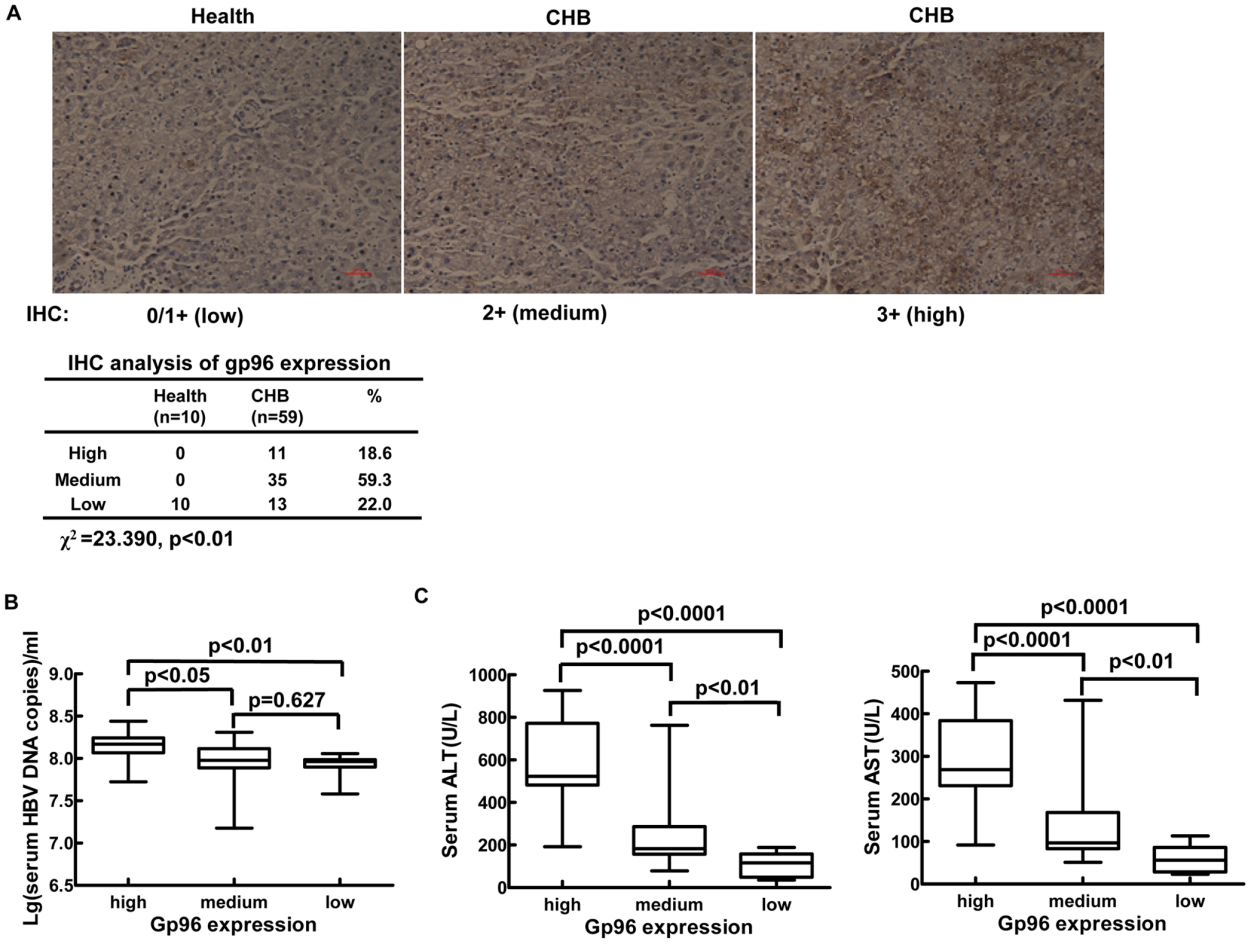
Gp96 expression in the liver is correlated with viral loads and serum ALT/AST levels in CHB patients. (A) IHC detection of gp96 expression in healthy and CHB liver tissues. Representative images indicating immunostaining intensities of 0/1+, 2+, and 3+ are shown. Statistical analysis of gp96 expression by Pearson’s χ test is presented in the lower panel. (B) Distribution of serum HBV DNA loads in CHB patients with low, medium, and high gp96 expression in the liver. (C) Distribution of serum ALT and AST levels in CHB patients with low, medium, and high gp96 expression. Student’s t test was used to determine P-values.

To address the clinical relevance of gp96 in HBV infection, we examined gp96 expression in 59 liver tissue samples from patients with CHB by IHC. Immunohistochemical staining of intracellular gp96 in liver tissue sections was qualitatively scored as 0/1+/2+/3+ according to the widely used scoring method for HER-2 IHC in breast cancer [27]. We found that 18.6% of CHB samples (11 of 59) had high gp96 expression, 59.3% (35 of 59) had medium gp96 expression, and only 22% (13 of 59) had low gp96 expression. In healthy controls, all samples displayed low gp96 expression (Fig. 6A). To investigate if active viral replication and HBV DNA loads correlate with gp96 up-regulation, we compared the serum HBV DNA levels among CHB patients with high, medium, and low gp96 expression. As shown in Fig. 6B, CHB patients with high gp96 expression had higher HBV DNA levels than those with medium or low gp96 expression (high vs. medium, 14.6±5. 87×10^7^/ml vs. 10.5±5.11×10^7^/ml, P<0.05; high vs. low, 14.6±5. 87×10^7^/ml vs. 8.67±2.25×10^7^/ml, P<0.01). There was a trend toward higher HBV DNA levels in CHB patients with medium gp96 expression than those with low expression, but this did not reach statistical significance. Spearman analysis revealed that gp96 expression positively correlates with HBV DNA loads (r = 0.32, P<0.05). In CHB patients with high gp96 expression, 73% (8 of 11) were also HBeAg-positive, whereas in those with medium and low gp96 expression, only 25% (12 of 48) were HBeAg-positive. Moreover, compared to patients with medium and low gp96 expression, those with high gp96 expression displayed much higher alanine aminotransferase (ALT) and aspartate aminotransferase (AST) levels (Fig. 6C). These findings suggest a reciprocal interaction between gp96 and HBV replication in CHB.

## Discussion

In this study, we investigated the reciprocal effect of gp96 on HBV replication. Our findings demonstrated that HBx-mediated NF-κB activation leads to increased gp96 transcription and expression, and increased gp96 can in turn promote HBV replication both *in vitro* and *in vivo*. In addition, we observed that elevated expression of gp96 is positively correlated with viral DNA loads in HBV-infected patients. Thus, our results provide new insights into the regulatory network of gp96 and suggest that up-regulated gp96 induced by HBV may contribute to viral persistence. Our data provide a basis for the potential application of gp96 as a prognostic marker or therapeutic target for HBV infection.

We identified a novel gp96 regulatory circuit involving HBx-NF-κB-gp96-HBV and suggested that this may contribute to viral persistence. To our knowledge, this is the first report of such HBV-induced up-regulation of host components that enhances viral expression and replication in CHB. HBV, which has a very small genome (∼3.2 kb in length) is notorious for its ability to usurp host cellular components and pathways to establish a persistent infection. However, the host factors that contribute to persistent viral infections are, in general, poorly understood. Indeed, to date, few studies have addressed this issue. Hösel *et al.* [31] show that IL-6 induced by HBV infection *in vitro* controls early viral expression and replication at the transcriptional level by inhibition of hepatocyte nuclear factor (HNF) 1α and HNF 4α. In another study, the conditional depletion of HNF 4α in HBV transgenic mice was found to result in loss of HBV transcription and replication, indicating that HNF 4α may increase the possibility of viral persistence [32]. From our current data, we conclude that gp96 induced in CHB functions as a stimulator of HBV, which may limit the host’s ability to resolve HBV infection.

Several lines of evidence demonstrate that accumulation of HBV large surface antigens and their various mutants can induce ER stress, which leads to the activation of GRP78 and gp96 expression [33], [34]. Li *et al*. [35], [36] also report that HBx can activate the ATF6 and IRE1-XBP1 pathways of the unfolded protein response (UPR), and as one of the target genes of the pathway, gp96 may be correspondingly activated. However, the mechanism by which gp96 is up-regulated during HBV infection remains elusive. In this report, we found that HBx induces gp96 expression at the level of transcription, which was dependent on the NF-κB binding site within the gp96 promoter and could be blocked by an NF-κB inhibitor (Fig. 2). Currently, we cannot exclude the possibility that HBx may also induce gp96 transcription and expression by targeting c-Myc or Sp1 within the gp96 promoter (Fig. 2A). Indeed, as can be seen in Fig. 2B, after mutation of the NF-κB binding site in the gp96 promoter, HBx treatment still increased the activity of the mutated gp96 promoter at the NF-κB site by about 50% compared to mock, indicating that the activation of c-Myc or/and Sp1 by HBx may also contribute to the elevated gp96 expression. Nevertheless, because HBx increased the activity of the wild type gp96 promoter by >1.5-fold, we deduce that HBx-mediated NF-κB activation plays a major role in elevated gp96 expression.

Currently, we cannot rule out the effect of hepatic inflammation on gp96 expression. Our current findings imply that up-regulation of gp96 may correlate with necro-inflammation (Fig. 6C). Indeed, treatment of Huh7 cells with IFN-α, IFN-γ, or TNF-α, which are usually elevated in CHB, significantly increased gp96 expression at the mRNA and protein levels in a dose-dependent manner (data not shown). These results demonstrated that a complex interplay of host and viral factors results in the up-regulation of gp96 in CHB.

Over-expression of gp96 by transfection of its expression vector led to a dramatic increase in HBV production, whereas knock-down of endogenous gp96 by RNAi suppressed HBV expression and replication *in vitro*. Moreover, in an HBV mouse model created by hydrodynamic injection, we found that over-expression of gp96 in the liver significantly enhances HBV expression and replication (Fig. 5). Thus, we suggest that gp96 supports HBV replication and expression. There are several possibilities to explain the effect. One possibility is that gp96 may promote pgRNA packaging via binding to HBV pol, which has been reported for the hsp90 [2]. The second possibility is that gp96 may regulate HBV expression by chaperoning the transcription factors that are essential for HBV transcription and/or assisting HBV antigens in folding and secretion. Alternatively, gp96 may up-regulate HBV expression by antagonizing cellular antiviral pathways such as the IFN pathway, which has been adapted by another chaperone calreticulin [37]. We observed that the HBeAg and viral particle levels in the cytoplasm and culture medium increased proportionally after gp96 transfection (data not shown), which ruled out the possibility that gp96 affects HBV production through regulation of virus secretion. Based on our observation that gp96 could affect HBV promoter activity and viral mRNA levels (Fig. 3 and Fig. 4), and importantly, the endoplasmic reticulum resident gp96 also exists in cell cytosol (data not shown), we consider that the cytosolic gp96 may chaperone transcription factors that are essential for HBV transcription, and thus enhance viral transcription and replication.

In conclusion, our work indicates the existence of a positive feedback loop between gp96 and HBV replication. HBx protein may directly induce gp96 expression through activation of NF-κB, and gp96 up-regulation in turn leads to enhanced HBV replication and production. Thus, this pattern of interaction may be advantageous for the virus, which can cause persistent infection with only a small amount of virions. In this context, the HBx-NF-κB-gp96-HBV circuit would therefore act in a novel way to enhance HBV infection, expanding the present knowledge of virus-host interactions and providing the potential to develop a gp96-based antiviral strategy to treat HBV infection.
